# Decellularized Extracellular Matrix for Cancer Research

**DOI:** 10.3390/ma12081311

**Published:** 2019-04-22

**Authors:** Takashi Hoshiba

**Affiliations:** 1 Biotechnology Group, Tokyo Metropolitan Industrial Technology Research Institute, Koto-ku, Tokyo 135-0064, Japan; hoshiba.takashi@iri-tokyo.jp; 2 Research Center for Functional Materials, National Institute for Materials Science, Tsukuba 305-0044, Ibaraki, Japan

**Keywords:** decellularization, extracellular matrix (ECM), cancer

## Abstract

Genetic mutation and alterations of intracellular signaling have been focused on to understand the mechanisms of oncogenesis and cancer progression. Currently, it is pointed out to consider cancer as tissues. The extracellular microenvironment, including the extracellular matrix (ECM), is important for the regulation of cancer cell behavior. To comprehensively investigate ECM roles in the regulation of cancer cell behavior, decellularized ECM (dECM) is now used as an in vitro ECM model. In this review, I classify dECM with respect to its sources and summarize the preparation and characterization methods for dECM. Additionally, the examples of cancer research using the dECM were introduced. Finally, future perspectives of cancer studies with dECM are described in the conclusions.

## 1. Introduction

Cancer is one of the leading causes of death, particularly in developed nations [1]. Thus, great efforts have been made to overcome this terrible disease. These endeavors have mainly focused on genetic mutations and intracellular signaling that lead to abnormal cellular behavior, such as loss of anchorage dependency, unlimited proliferation, promotion of migration, and increased chemoresistance [2,3,4,5]. Recently, it has been indicated that cancer should be considered as a tissue to more fully understand the mechanisms of cancer cell behaviors [1,6]. In cancer tissues, there are noncancerous cells (e.g., endothelial cells and fibroblasts), growth factors, and extracellular matrix (ECM) [1,6]. In particular, ECM is key to regulating various cell behaviors [6,7,8].

ECM is composed of various types of proteins and carbohydrates [9], which are assembled to form special architectures that can interact with cells via cell surface receptors, such as integrins [10,11]. ECM plays pivotal roles in regulating cell behaviors, such as cell adhesion, survival, proliferation, morphogenesis, migration, and responses to growth factors, via various modes [10,11,12,13]. Thus, ECM likely plays important roles in the initiation and progression of cancer. To understand the roles of ECM in cancer, researchers have focused on single or multiple ECM molecules and have examined the effects of these molecules on cell behavior utilizing genetically mutated cells/animals and substrates coated with ECM molecules [14,15,16,17]. However, ECM is the assembly of many molecules, and assembled ECM molecules activate multiple intracellular signaling pathways [9,10,11,12,18]. Finally, activated intracellular signals are orchestrated within cells to exhibit specific cellular behaviors. Thus, it is necessary to comprehensively investigate the roles of ECM in cancer initiation and progression with respect to the assembly of ECM molecules.

Reconstituted ECM is useful for comprehensive studies examining roles of the ECM in cancer initiation and progression. In the last decade, decellularization has been largely developed to obtain reconstituted ECM in vitro, which is known as the decellularized ECM (dECM) [19,20,21,22,23]. In this review, I summarize the preparation and characterization of dECM after describing why dECM is necessary and important for reconstituting ECM in vitro. In addition, examples of the studies using dECM to examine cancer initiation and progression will be introduced. Finally, we will summarize the problems that remain to be solved in the future.

## 2. dECM as Reconstituted Native ECM In Vitro

### 2.1. ECM Composition and Structure

There are approximately 300 types of ECM proteins and carbohydrates [24]. These ECM molecules can be categorized into three types: Collagens, proteoglycans, and glycoproteins [9]. Collagens act as structural support for the ECM, and at least 19 types of collagen have been reported to date [25]. Proteoglycans, such as perlecan and aggrecan, are composed of two parts (i.e., core proteins and glycosaminoglycans (GAGs)). These proteoglycans store abundant water molecules in GAG chains and bind various soluble factors to control their availabilities [9,26,27]. Free GAGs, such as hyaluronan, also exist in ECM. Glycoproteins support cell adhesion via various receptors on the cell membrane [9,10]. There are approximately 200 types of glycoproteins, such as fibronectin, laminins, vitronectin, and fibrinogen [9,24]. These ECM molecules interact with the same/different molecules to form fibrous or meshwork macromolecules and further assemble into special architecture like basement membrane [28].

### 2.2. ECM in Cancer Pathology

Molecular types in assembled ECM differ among tissue types, developmental stages, and pathological conditions [18,24,29,30,31,32]. In cancer tissues, the ECM composition differs based on the tissue type and malignant levels [24,31,32]. Also, the ECM microstructure might also vary according to the tissue type and malignant level. Indeed, the ECM composition and ECM molecules/fragments can be used as the indicators of cancer malignancy and to estimate cancer prognosis. It seemed that ECM and ECM remodeling play important roles in cancer initiation and progression. Thus, the researches have been extensively performed to unveil the role of ECM molecules in cancer initiation and progression [31,33,34]. However, there are few researches to investigate the roles of assembled ECM molecules in cancer pathogenesis comprehensively. Also, the researches focusing on ECM remodeling are very few. To understand the roles of ECM in cancer initiation and progression, the compositional and structural dynamics of the ECM should be considered using assembled ECM molecules. In vitro ECM models will be helpful for this purpose.

### 2.3. Decellularized ECM (dECM)

As described above, ECM is composed of many types of ECM molecules, and assembled ECM molecules differ among tissue types and malignant levels. It is very difficult to reconstitute ECM in vitro using conventional chemical and physical methods. Native ECM exists in the tissues and organs of the body. Furthermore, ECM is contained in cultured cells/ECM constructs (i.e., tissues and organs that are reconstructed by cultured cells) that are formed by tissue engineering approaches. Thus, reconstituting native ECM is possible by removing the cellular components from these cells/ECM constructs (i.e., native or regenerated tissues and organs). The decellularization technique is used for removing cellular components from the cell/ECM constructs, and the resulting remnant ECM is known as dECM.

#### 2.3.1. Comparison of Sources for dECM

There are two main sources for obtaining dECM: One is from the native tissues and organs of the body, and the other is from regenerated tissues and organs that are constructed from cultured cells [19,20,21,22,23]. Thus, dECM is usually categorized as tissue/organ-derived dECM and cultured cell-derived dECM. There are several advantages and disadvantages to each approach (Table 1). It is expected that tissue/organ-derived dECM possesses a similar composition and microstructure to the native ECM. In contrast, the composition and microstructure of cultured cell-derived dECM can be easily altered by the culture conditions (e.g., culture media, initial substrates, culture periods, cell types, and passage numbers) [35,36,37,38,39,40,41]. Thus, the similarities between the tissue/organ-derived dECM and native ECM are the greatest advantages for culturing cell-derived dECM. However, cultured cell-derived dECM can reconstitute native ECM in limited regions, such as a stem cell niche, if it is well prepared [42,43]. The reconstitution of ECM in limited regions is difficult for the tissue/organ-derived dECM because these regions are difficult to identify and isolate. In addition, the supply of tissue and organs for preparing dECM is limited compared to cultured cells. It is often difficult to analyze intracellular signaling in tissue/organ-derived dECM using large-scaled cell and molecular biology methods due to low available sample numbers. For cancer research, working with tissue/organ-derived dECM is challenging because the composition and microstructure of cancerous ECM might be heterogeneous among patients. Actually, the composition and microstructure of native ECMs are different, even in patients diagnosed with the same cancer. For this reason, the batch-to-batch variation is extensive in tissue/organ-derived dECM. Thus, selecting dECM sources for cancer research requires careful consideration.

#### 2.3.2. Preparation

The most important aspect of dECM preparation is decellularization, because these methods strongly influence the composition and microstructure of dECM [44,45,46]. Many methods have been previously reported and are primarily categorized into three types: Chemical, physical, and enzymatic. Detergents, such as sodium deoxysulfate (SDS), Triton X-100, and sodium deoxycholate (SDC), are typically used for chemical methods. These detergents solubilize cytoplasmic and nuclear lipid membranes and proteins for efficient decellularization, but they tend to disrupt the ECM microstructure and results in the loss of ECM components to some degree. In addition to these detergents, alkaline and acid components are also used for chemical decellularization methods because they solubilize cytoplasmic components and disrupt nucleic acids. However, these reagents can also reduce GAGs. For physical decellularization, freeze-thaw cycles are used to disrupt the cell membrane, but this method can also disrupt or fracture the ECM structure. Trypsin and other proteinases are sometimes used for enzymatic decellularization, but prolonged exposure to proteinases leads to the degradation of proteins and thus disrupts the ECM structure. For nucleic acid removal, nucleases are often used, which remove nucleic acids effectively but cannot remove any of the cytosolic components. As described above, these methods all have advantages and disadvantages compared to each other. Thus, they are usually used in combination to overcome their disadvantages.

In addition to decellularization methods, other factors can alter the composition and microstructure of cultured cell-derived dECM, and these alterations result in different functionalities [21]. Culture conditions, such as media composition and cocultured cells, can alter the composition and microstructure of the cultured cell-derived dECM and the formation speed of ECM beneath the cells [35,36,37]. Initial substrates can also alter the ECM composition and microstructure. Furthermore, substrates can change the formation speed and biological activity of ECM formed beneath cells [38,39]. Moreover, substrates can be removed to obtain the scaffold-free cultured cell-derived dECM when degradable substrates are used [47]. Cell type and passage number are critical to determine the composition and microstructure of dECM because expression patterns of ECM change based on cell type and passage number [40,41].

Sometimes, the modification of dECM is required for cancer research. Enzymes are typically used to remove specific molecules from the dECM. To add specific molecules, the dECM is immersed in a solution with the target molecules. The mechanical properties of the ECM are also important for cancer research, and mechanical properties can be changed by crosslinking with genipin [48].

Solubilization is sometimes performed, particularly for tissue/organ-derived dECM [49,50]. The obtained dECM is treated with acidic pepsin for solubilization, and solubilized dECM can be gelled by neutralization and warming. The solubilization of dECM is a good method to obtain more homogenous samples. However, the microstructure of dECM may be compromised by this method.

#### 2.3.3. Characterization

Characterization is important to confirm whether the expected dECM is obtained (Table 2). First, it is necessary to examine whether cellular components were removed from the dECM. For this purpose, a cell nuclear component (i.e., DNA) is typically detected by staining for cell nuclei (e.g., hematoxylin and Hoechst 33258 staining) or by measuring DNA content. Additional intracellular components are often detected using histochemical methods (e.g., staining with fluorescent-labeled phalloidin for fibrous actin detection and immunocytochemistry of cytosolic proteins).

For the compositional characterization of dECM, histological methods have been used. Eosin staining can detect non-nucleic components. Alcian blue and toluidine blue stainings can detect GAGs. Sirius red and Azan stainings can detect collagens. In addition to these histochemical methods, immunochemical methods with specific antibodies are typically used to detect specific ECM proteins. Also, specific GAGs can be often detected with lectins. Moreover, mass spectrometry has been utilized for dECM compositional analysis exhaustively.

Structural analyses of dECM are performed using the scanning electron microscopy (SEM) and transmission electron microscopy (TEM). In particular, TEM can be used to assess the basement membrane formation because lamina densa is observed as an electron dense region [37]. Furthermore, the fast Fourier transform analysis can assess fibril alignment [51]. Recently, Li et al. reported the peptides that can hybridize with denatured collagen triple helix, and denatured collagens can be visualized with these peptides [52,53].

## 3. Examples of dECM Utilized for Cancer Research

In cancer research, the ECM is one of the tumor tissue elements that are studied to understand the molecular mechanisms of cancer cell behavior. It has been extensively examined to clarify the relationship between single ECM molecules and cancer prognosis to identify new diagnostic cancer markers using histological methods [54,55]. For the purpose of detailed investigations, genetically mutated animals and cells have been used [14,16]. Additionally, culture substrates coated with single or multiple proteins have been used to examine cellular responses [15,17]. These studies have achieved many important insights, ranging from the observation of cancer cell behavior to intracellular signaling activation. However, the ECM comprises the assembly of many different molecules as described above. Assembled ECM molecules can activate multiple intracellular signaling pathways, and these signals are orchestrated to regulate cellular behavior. Thus, comprehensive investigation of the effects of assembled ECM molecules (i.e., native ECM) on cell behaviors is required to fully understand the roles of the ECM. dECM can provide a suitable platform for comprehensive examination of the ECM in cancer biology. Thus, dECM has been developed and utilized for cancer research (Table 3). dECM used in the researches can be classified into two types by the combination between the sources of cultured cells and dECM. When the sources of dECM and the cells cultured on dECM are the same, dECM can be used as models of ECM at primary sites. If the sources of dECM and the cells cultured on dECM are different, dECM can be used as models of ECM at metastatic sites. In this chapter, we introduce the recent cancer researches with dECM, classified into the above two types.

### 3.1. Cell Behaviors in/on dECM as ECM Models at Primary Sites

Many dECMs have been prepared for cancer research from normal and diseased tissues that were used for the culture of cancer cell lines originating from the same tissues. Culture models using cells derived from the same tissues of dECM might represent useful models to examine cell behavior in primary cancer tissues.

#### 3.1.1. dECM Derived from Normal Tissues

Mishra et al. prepared the dECM that was derived from normal rat lung whole tissues and were cultured with human A549, H460, and H1299 lung cancer cell lines [56]. Cancer cells proliferated and formed a pattern with pathologic appearances that were similar to those of the original cancer tissues in normal whole lung dECM. Dunne et al. prepared dECM that was derived from normal human adipose tissue for breast cancer research [58]. They claimed that breast cancer cells are surrounded by adipose tissues and that adipose tissue-derived dECM mimics ECM in breast cancer. They cultured MCF-7, BT474, and SKBR3 breast cancer cell lines in this adipose tissue-derived dECM, and proliferation was observed in these cells. Moreover, the expression of *CDH1* (E-cadherin) decreased, and the expression of *CDH2* (N-cadherin) and *VIM* (vimentin) increased in the dECM compared to 2D culture with plastic dishes and culture on Matrigel, which is a crude extract of the basement membrane formed by Engelbreth-Holm-Swarm sarcomas. This result suggests that dECM promotes the epithelial-mesenchymal transition (EMT). Finally, they assessed chemoresistance towards doxorubicin and demonstrated that chemoresistance was increased in the dECM via the activation of epidermal growth factor receptor and Akt. The dECM derived from normal liver was also developed to study cancer migration [59]. Sun et al. prepared normal liver-derived dECM and alginate hybrid gel beads and cultured a hepatocellular carcinoma HCCLM3 cell line in the beads. Enzymes that are related to cancer metastasis via the ECM degradation were subsequently examined. They reported that the urokinase-type plasminogen activator (uPA) production and matrix metalloproteinase (MMP)-2 and MMP-9 activities were increased in dECM containing beads. In contrast to these ECM degrading enzymes, plasminogen activator inhibitor-1 (PAI-1) production was reduced in the beads. These results suggest that cancer cells promote their own migration.

#### 3.1.2. dECM Derived from Cancer Tissues

The research described above was performed using dECM that was derived from normal tissues. However, the ECM composition is reportedly different between normal and cancerous tissues. Thus, it is expected that the dECM that is derived from cancerous tissues is more suitable for examining the role of cancerous ECM in the regulation of cancer cell behavior at primary sites. Liu et al. prepared dECM from human breast cancer tissues that were cultured with the breast cancer MCF-7 cell line [62]. In the dECM, CDH1 expression decreased during culture, while the expression of the EMT genes *VIM*, *ZEB1*, and *SNAI1* increased. These results suggested that EMT is promoted by cancer-derived dECM. Moreover, they demonstrated that the resistance against 5-fluorouracil (5-FU) increased in the dECM compared with cultures lacking dECM. Interestingly, the expression of genes encoding stem cell markers (Oct4 and Sox2) and a breast cancer stem cell marker (CD49f) was maintained in the dECM when cells were treated with 5-FU. Thus, they concluded that dECM derived from breast cancer tissues is suitable for breast cancer research. Koh et al. prepared dECM derived from glioblastoma multiforme [63]. They solubilized the dECM and mixed it with type I collagen for culture in 3D gel. Invasion of glioblastoma cells derived from patients was examined quantitatively in the gel. The invasion of glioblastoma cells was accelerated via the morphological change on the cancer-derived dECM. Moreover, the expression of *MMP9* and hyaluronan synthases (HASs) (*HAS1*, *HAS2*, and *HAS3*) were increased in dECM-containing gels, which suggeststhat cell invasion was increased via the ECM degradation and hyaluronan deposition, which promotes cell invasion. Indeed, cell invasion in the gels was inhibited by the addition of either the MMP or HAS inhibitor.

#### 3.1.3. Comparison of Cell Behavior in dECM Derived from Normal and Cancer Tissues

Comparative studies of cell behavior in normal and cancerous tissue-derived dECM were also performed to determine the roles of cancerous ECM. Jin et al. compared the behavior of the MCF-7 cell line in dECM that was derived from normal and cancerous breast tissues [68]. They demonstrated that the proliferation of MCF-7 cells was decreased in cancerous dECM compared with normal dECM. In contrast, cancerous dECM promoted angiogenesis, EMT responses and MMP-9 production compared to normal dECM. These results suggest that the cancerous ECM plays different roles in the regulation of cancer cell behavior, compared to the normal ECM. Miyauchi et al. prepared dECM from normal and fibrotic livers because liver fibrosis is the primary risk factor for hepatocellular carcinoma, allowing the comparison of cell behaviors between cultures in normal and fibrotic liver dECM [70]. They cultured hepatocellular carcinoma HuH7 and HLF cell lines in these dECM samples, and cells exhibited faster proliferation and stronger EMT responses in the fibrotic liver dECM than in normal dECM. In addition, the authors suggested that these responses were due to the excess activation of integrin signaling.

Recently, inflammatory responses were shown to promote cancer cell invasion [78]. Thus, Pinto et al. prepared dECM from normal and cancerous colorectal tissues with cultured human monocytes in each dECM [66]. They demonstrated that monocyte differentiation was promoted in cancerous dECM, and C–C motif chemokine ligand 18 (CCL18) production was increased in cancerous dECM, promoting cancer cell invasion.

To identify key molecules that promote cancerous behavior of cancer cells, mass spectrometry has been applied. Piccoli et al. demonstrated the promotion of cell migration and angiogenesis in colorectal cancerous dECM compared with normal dECM [67]. Mass spectroscopy was applied to compare ECM components between normal and cancerous dECM to identify the precise components promoting angiogenesis. Ultimately, they proposed DEFA3 as a candidate factor that promotes angiogenesis.

All studies described above used tissue/organ-derived dECM. In addition to the studies using tissue/organ-derived dECM, cultured cell-derived dECM is also used for cancer research. Cukierman and colleagues prepared 3-dimensional (3D) dECM from the murine normal fibroblast NIH-3T3 cell line and cancer-associated murine fibroblasts [73,74]. They focused on fibroblasts as an ECM source in tissues because fibroblasts produce abundant ECM molecules. They compared cell behaviors in 3D dECM produced by normal and cancer-associated fibroblasts with respect to cellular phenotypes and intracellular signaling. They cultured breast cancer MCF-7 and MDA-MB-231 cell lines and a benign breast tumor MCF-10A cell line in 3D dECM. Particularly, MDA-MB-231 cells exhibited increased spindle shapes in cancer-associated fibroblast-derived dECM via Akt activation.

As described above, many dECM types have been proposed to examine cell behaviors to understand the roles of ECM in cancer. dECMs were prepared from normal and cancerous tissues, and cells exhibited different behaviors between normal and cancerous dECM due to compositional and structural differences. Overall, cancerous dECM seem to promote cell migration, angiogenesis, and EMT responses. Thus, it might be suitable to use cancerous dECM as a model of native cancerous ECM for investigating the roles of ECM in cancer at primary sites.

### 3.2. Mechanism Analysis of Chemoresistance

Chemoresistance is one of largest barriers to cancer therapy. Clinically, chemoresistance increases with cancer progression [79]. Therefore, several studies have examined mechanisms of chemoresistance at the molecular level using dECM. For the analysis of chemoresistance mechanisms at the molecular level, cultured cell-derived dECM has often been used.

Serebriiskii et al. reported that normal fibroblast-derived 3D dECM tended to increase the resistance of five types of cancer cell lines against Taxol via an integrin β1-dependent, and focal adhesion kinase- (FAK) and Akt-independent manner compared to plastic substrates [73].

Berrier et al. prepared dECM from cultured metastatic cancer cells (HN12) that were isolated from the lymph nodes of a patient with tongue cancer. They cultured HN12 cells on cultured HN12 cell-derived dECM and ECM protein-coated substrates [71] and observed that the resistance against cisplatin increased on cultured HN12 cell-derived dECM. In addition, they demonstrated that the increased cisplatin resistance was due to interaction via integrins following intracellular signaling activation, particularly of talin, FAK, and nuclear factor (NF)-κB [71,72].

**Figure 1:**
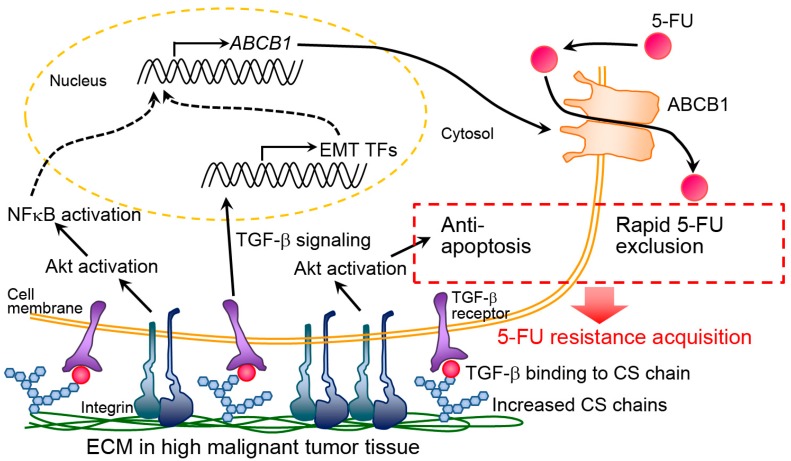
Putative molecular mechanism of chemoresistance acquisition by highly malignant extracellular matrix (ECM). CS and TF indicate chondroitin sulfate and transcription factor, respectively. This figure is reproduced from [77] with the permission of Elsevier.

Furthermore, Hoshiba et al. prepared dECM derived from cultured cells with different malignant levels known as “staged tumorigenesis-mimicking matrices” [39,75]. We prepared staged tumorigenesis-mimicking matrices through the culture of breast and colorectal cells and found that the resistance of breast and colorectal cancer cells against 5-FU increased in response to dECM that was derived from cells with the highest malignant level (i.e., highly invasive cells). Specifically, 5-FU resistance mechanisms were investigated in colorectal cancer [76,77]. We prepared staged colorectal tumorigenesis-mimicking matrices using the culture of invasive HT-29 cells (highly malignant dECM), noninvasive SW480 cells (low malignant dECM), and normal CCD-841-CoN cells (normal dECM). HT-29 cells were also cultured on staged tumorigenesis-mimicking matrices. On highly malignant dECM, HT-29 cells expressed the drug-efflux transporter *ABCB1* at the highest levels among the staged tumorigenesis-mimicking matrices [76]. When HT-29 cells were exposed to 5-FU, the cells underwent EMT and increased *ABCB1* expression via the TGF-β signaling pathway. Highly malignant dECM possessed abundant chondroitin sulfate chains, which can interact with TGF-β and efficiently presented it to cells. Thus, highly malignant dECM increased the *ABCB1* expression via intracellular signal activation by TGF-β, which was efficiently presented to cells via the binding to chondroitin sulfate [77]. Further, Akt activation partially contributed to the 5-FU resistance in HT-29 cells [76] (Figure 1).

For new anticancer drug development, in vitro culture systems for drug testing are very important given the concerns regarding animal welfare and cost saving measures. However, cancer cells do not maintain chemoresistance during conventional in vitro culture. In contrast, dECM increases the chemoresistance of cancer cells in vitro and is expected to induce the responses similar to in vivo. Hence, dECM is expected to be a suitable cell culture substrate for pharmacological and pharmacokinetic analyses.

dECM is a powerful platform to examine cancer cell behavior. Thus, both tissue/organ-derived dECM and cultured cell-derived dECM are used. In particular, cultured cell-derived dECM, as opposed to tissue/organ-derived dECM, is likely to be used for analysis of chemoresistance mechanisms. Analysis at the molecular level is required to elucidate chemoresistance mechanisms. It seemed difficult for tissue/organ-derived dECM to analyze in large-scaled systems with small batch-to-batch differences, compared with cultured cell-derived dECM. Fewer sample numbers and large batch-to-batch differences might make this modality more difficult to use for molecular mechanism analyses. Thus, cultured cell-derived dECM seems a preferable substrate for the analysis of chemoresistance mechanisms due to its abundant supply with reduced batch-to-batch variation. However, it should be considered whether cultured cell-derived dECM truly mimics native ECM in cancer tissues.

### 3.3. Cancer Cell Colonization at Metastatic Sites

Cancer metastasis is one of the largest problems in cancer therapy. Improved understanding of metastatic mechanisms is expected to provide important information to inhibit metastasis. The process of cancer metastasis to distant organs is not random but rather is predetermined, as indicated by Paget’s “seed and soil” hypothesis [80]. This hypothesis suggests that some distant organs provide a more suitable extracellular microenvironment (soil) than other organs for promoting colonization of certain cancer cells (seed). Thus, the extracellular microenvironment at metastatic sites seems crucial for cancer cell colonization and metastasis. ECM is one of the most important factors in the extracellular microenvironment; thus, it is hypothesized that ECM somewhat determines the fate of metastatic cancer cells. dECM has begun to be used for verification of this “seed and soil” hypothesis by examining cancer cell colonization. For this verification, it is examined how cancer cells interact with ECM in metastatic sites using dECM derived from distant target tissues.

Xiong et al. compared proliferation in dECM that was derived from normal lung tissue between invasive breast cancer MDA-MB-231 cells and noninvasive breast cancer MCF-7 cells [57]. As expected, MDA-MB-231 cells invaded and colonized the dECM. In contrast, few MCF-7 cells invaded and proliferated in the dECM, and some cells underwent apoptosis. MDA-MB-231 cells underwent EMT, but MCF-7 cells did not. When *ZEB1* expression decreased through siRNA to recover MDA-MB-231 cells from EMT, the cells that recovered from EMT were unable to colonize the dECM. These results suggest that EMT is important for colonization in the extracellular microenvironment in distal organs.

Tian et al. prepared dECM derived from normal liver and lung tissues and cultured colorectal cancer cells (HT-29, SW480 and Caco2 cells) in these dECMs [59]. In dECM derived from liver, cells exhibited comparable morphology to liver metastases found in vivo. In addition, cells harvested from culture in these dECMs possessed higher metastatic potential compared to cells that were not cultured in dECM. These results suggest that cells are educated by the dECM to obtain increased metastatic potential.

A study to identify the key components of metastasis was also performed using dECM and mass spectrometry [69]. Aguado et al. demonstrated that breast cancer 4T1 and LM2-4 cells adhered and colonized on dECMs derived from both cancerous tissues derived from the liver and lung to a greater extent than those from corresponding healthy tissues. To identify components that promote adhesion and colonization of these cells, proteins in dECM derived from both cancer and healthy tissues were compared by the mass spectrometry. Finally, the authors proposed that myeloperoxidase promoted cancer cell colonization. As reported in these studies, dECM enables verification of the “seed and soil” hypothesis, which is difficult to investigate using conventional biological methods and materials.

## 4. Future Perspectives

### 4.1. Availability of dECM as In Vitro ECM Models in Cancer

Previous ECM studies have focused on only single or multiple ECM molecules and have examined cell behavior and intracellular signaling. However, ECM is the assembly of multiple ECM molecules that activate many intracellular signaling pathways orchestrated to regulate cell behavior. Thus, cell behavior might be different between single or multiple ECM molecules and assemblies of ECM molecules (i.e., native ECM). dECM can provide the assembly of many ECM molecules, which mimics native ECM. dECM enables the examination of cell behavior when cells interact with the assembly of ECM molecules. Indeed, dECM has been used in comprehensive studies for stem cell biology [20], thereby providing us with new insights. Similar to stem cell biology studies, dECM has begun to be used for various cancer studies as described above. It is expected that these investigations will provide new insight into cancer research in the future.

However, it is worth noting that cancer is a heterogeneous disease, and ECM compositions are dependent upon conditions (e.g., malignant levels) [31,32]. This heterogeneity makes it difficult to examine the roles of the ECM using dECM. For this heterogeneity, the cancer phenotypes of dECM sources should be clearly described to systematically understand and integrate results. For example, clinical cancer stage, invasive and metastatic ability, and molecular markers of cancer tissues might be good indicators of cancer phenotype. Cell name for the preparation should also be addressed for the cultured cell-derived dECM.

### 4.2. Proper Selection of dECM Preparation Methods

Tissue/organ-derived dECM might provide more suitable in vitro ECM models than cultured cell-derived dECM because tissue/organ-derived dECM easily retain their original compositions and structures. Thus, tissue/organ-derived dECM appear to be suitable to examine cancer cell behaviors at both primary and metastatic sites. However, cultured cell-derived dECM appears to be preferable to tissue/organ-derived dECM for large scale analyses of intracellular signaling activated by ECM because cultured cell-derived dECM can be obtained in abundance with small batch-to-batch differences (Table 1). Thus, various molecular biology-based techniques can be easily applied for analyses with cultured cell-derived dECM. For the same reasons, cultured cell-derived dECM is preferable to tissue/organ-derived dECM for pharmacological and pharmacokinetic testing. However, it should be checked whether cultured cell-derived dECM is similar to native ECM from the viewpoints of cancer phenotypes. Additionally, it should be confirmed whether expected drug responses are obtained with cultured cell-derived dECM.

Decellularization methods are also an important factor to prepare dECM for cancer research because these methods strongly influence the dECM composition and structure, which determine cell behavior. Thus, the dECM composition and structure should be characterized. Mass spectrometry has begun to be applied to identify key ECM components that induce specific cell behaviors [67,69]. This proteomic analysis might pave the way for new cancer therapies. However, if improper methods are selected to decellularize and ECM components are lost from the dECM as a result, then it is possible to omit ECM components that truly determine cancer cell behavior. Decellularization methods should be selected carefully due to these reasons.

### 4.3. Possibility of Contributions to Cancer Therapies

In this review, some examples of dECM used in cancer research were summarized. In addition to basic research, dECM might contribute to cancer therapy. A representative example is pharmacological and pharmacokinetic analyses using dECM, as described in detail above. It may be possible that dECM derived from normal tissues suppresses cancer cell proliferation. Indeed, Hoshiba et al. demonstrated that cultured normal mammary gland cell-derived dECM suppresses breast cancer proliferation [75]. In addition, Xiong et al. demonstrated that normal lung tissue-derived dECM induced apoptosis of MCF-7 cells that do not undergo EMT [57]. These results suggest that normal ECM tissue possesses components that can suppress cancer progression. If these key components are identified by proteomic analysis with mass spectrometry, they will be candidates for anticancer drugs. However, only a few reports have suggested this possibility thus far, and further investigations are required.

## 5. Conclusions

In the last few decades, the decellularization technique has been well developed. Currently, dECM has begun to be used for cancer biology research. However, there are several points to be considered for its use. Despite its shortcomings, dECM will provide new insights into the role of ECM in oncogenesis and cancer progression. Furthermore, dECM represents a culture substrate for pharmacological and pharmacokinetic analyses to develop novel anticancer drugs. Moreover, proteomic analysis of dECM will lead to the identification of candidates for anticancer drugs. Thus, dECM is a promising native ECM model for comprehensively investigating the roles of ECM in the oncogenesis and cancer progression to contribute to cancer therapy.

## Figures and Tables

**Table 1:** Comparison of decellularized extracellular matrix (dECM) sources for cancer research.

dECM Type	Advantages	Disadvantages
Tissue/organ-derived dECM	-Similar to native ECM composition and structure	-Limited ECM sources -Difficult for large-scale in vitro analyses -Large batch-to-batch differences due to cancer heterogeneity
Cultured cell-derived dECM	-Possible for large-scale in vitro analyses	-Difficult to prepare dECM that completely mimics native ECM composition and structure

**Table 2:** Frequently used characterization methods of dECM.

Purposes	Principle	Methods
Confirmation of cell removal	DNA/cell nuclei detection	-Staining with hematoxylin and Hoechst 33258 -DNA content measurement
	Intracellular protein detection	-Actin staining with fluorescent-labeled phalloidin -Immunocytochemistry of cytosolic proteins
Compositional analysis	Detection of non-nucleic components	-Eosin staining
	GAGs detection	-Alcian blue and toluidine blue stainings
	Collagens detection	-Sirius red and azan stainings
	Specific proteins/carbohydrates detection	-Immunohistochemical analysis with antibodies -Staining with lectins
	Proteomics (exhaustive research)	-Mass spectrometry
Structural analysis	Structure observation	-SEM
	Basement membrane detection	-TEM
	Fibril alignment	-fast Fourier transform analysis

**Table 3:** Partial list of dECM utilized for cancer research.

dECM Type	Malignancy of dECM Source	Tissue/Cell of dECM Origin	Cells Cultured on dECM	Results	Reference
Tissue/organ- derived	Normal	Lung	Lung cancer A549, H460, H1299 cells	-Developed pattern of growth similar with original human lung cancer.	[56]
			Breast cancer MDA-MB-231 and MCF-7 cells	-MDA-MB-231 cells undergone EMT can proliferation. -MCF-7 cells not undergone EMT died by apoptosis.	[57]
		Adipose tissue	Breast cancer MCF-7, BT474, SKBR3 cells	-Proliferation, underwent EMT, and increased invasion. -Increased chemoresistance via Akt.	[58]
		Liver	Hepatocellular carcinoma, HCCLM3 cells	-Increased uPA production and MMP-2 activity. -Decreased PAI-1 production.	[59]
			Hepatocellular carcinoma, HepG2 cells	-Increased expression of genes relating to hepatic functions.	[60]
		Liver and lung	Colorectal cancer HT-29, Caco2 and SW480 cells	-Exhibited morphology and gene expression pattern similar with metastatic sites of colorectal cancer. -The cells educated by dECM acquire metastatic ability.	[61]
	Cancer	Mammary grand	Breast cancer MCF-7 cells	-Underwent EMT, increased stem cell marker expression and chemoresistance.	[62]
		Glioblastoma	Isolated glioblastoma cells	-Increased invasive ability via HAS gene expression.	[63]
		A549-derived lung cancer	Breast cancer MCF-7 cells	-Cell proliferation. -Increased IL-8, bFGF, and VEGF production.	[64]
	Normal and cancer	Colon	Colorectal cancer SW620, SW480, HCT116 cells, normal lung fibroblasts, endothelial colony forming cells	-Increased angiogenesis and cancer cell proliferation in cancer tissue-derived dECM.	[65]
			Isolated monocytes	-Promoted monocyte differentiation and CCL18 production to accelerate cancer cell invasion.	[66]
			Colorectal cancer HT-29 cells	-Increased IL-8 production in cancer tissue-derived dECM.	[67]
		Breast	Breast cancer MCF-7 cells	-Suppressed proliferation, EMT and angiogenic gene expression and increased apoptosis in normal tissue-derived dECM. -Promoted MMP-9 production, proliferation, EMT, and angiogenic gene expression and suppressed apoptosis in cancer tissue-derived dECM.	[68]
		Lung and liver	Breast cancer LM2-4 and 4T1 cells	-Promoted cell adhesion and colonization in cancer tissue-derived dECM.	[69]
	Normal and fibrosis	Liver	Hepatocellular carcinoma HLF and HuH7 cells	-Promoted proliferation. -Promoted EMT via integrin-FAK signaling.	[70]
Cultured-cell-derived	Cancer	Tongue (Oral carcinoma HN12 cells)	Oral carcinoma HN12 cells	-Increased chemoresistance via talin, FAK, and NF-κB-mediated signals	[71,72]
	Normal	Fibroblasts (NIH-3T3)	Various cancer and benign cells (HCT116, NCI-H460, PA-1, COLO 205, PANC-1, MCF-7, SW620, HCT116/p53-, HS 578T, PA1/E6, MCF-10A	-Increased chemoresistance via integrin β1-dependent survival signal.	[73]
	Normal and cancer	Fibroblasts (NIH-3T3 cells and cancer associated fibroblasts)	Breast cancer MDA-MB-231, MCF-7, and MCF-10A cells	-Activated PI3K-Akt signaling via integrin β1. -Changed morphology and cell migration behaviors	[74]
	Benign tumor and cancer	Breast (MDA-MB-231, MCF-7, and MCF-10A cells)	Breast cancer MDA-MB-231, MCF-7, and MCF-10A cells	-Promoted proliferation on invasive MDA-MB-231 cell-derived dECM. -Suppressed proliferation on benign MCF-10A cell-derived dECM. -Increased chemoresistance on invasive MDA-MB-231 cell-derived dECM.	[75]
	Normal and cancer	Colon (HT-29, SW480, CCD-841-CoN cells)	Colon cancer HT-29 and SW480 cells	-Increased chemoresistance on invasive HT-29 cell-derived dECM via Akt activation and *ABCB1* upregulation. -Promoted EMT on invasive HT-29 cell-derived dECM via TGF-β signaling.	[39,76,77]
